# Attitudes towards Antibiotic Prescription and Antimicrobial Resistance Awareness among Italian Dentists: What Are the Milestones?

**DOI:** 10.3390/healthcare10081585

**Published:** 2022-08-21

**Authors:** Francesco D’Ambrosio, Federica Di Spirito, Alessandra Amato, Mario Caggiano, Roberto Lo Giudice, Stefano Martina

**Affiliations:** 1 Department of Medicine, Surgery and Dentistry, University of Salerno, 84084 Fisciano, Salerno, Italy; 2 Department of Neuroscience, Reproductive Science and Dentistry, University of Naples Federico II, 80138 Naples, Campania, Italy; 3 Department of Human Pathology in Adulthood and Childhood “G. Barresi”, University Hospital “G. Martino” of Messina, 98123 Messina, Sicily, Italy

**Keywords:** antibiotics, antimicrobial resistance, antibiotic stewardship, dentistry, dentist

## Abstract

Antimicrobial resistance is a growing phenomenon, often associated with the improper prescription of antibiotics, prescribed by those who do not follow the guidelines for antibiotic stewardship. The aim of this study was to evaluate the current attitude towards antibiotic prescribing and antimicrobial resistance awareness among Italian dentists. An online questionnaire was distributed to Italian dentists from September to December 2021. The questionnaire was composed of three main sections. The first part was focused on demographic characteristics, the second part on prescription habits, and the third part on the dentists’ knowledge regarding the phenomenon of antimicrobial resistance. A chi-square test was used to find associations between different variables. The level of significance was set at *p* < 0.05. A total of 382 dentists completed the questionnaire. The main reasons for antibiotic prescribing were for abscesses (39.6%), extractions (24.5%), and pulpitis (14.1%). The majority of participants (85.3%) reported prescribing macrolides in the case of a penicillin allergy. Most dentists (98.9%) were aware of the antimicrobial resistance phenomenon, but only 7.4% of them consulted the guidelines for prescribing antibiotics. This study demonstrates that the same trend exists in Italy as in other countries in terms of the high prevalence of misuse and overuse of antibiotics, and that Italian dentists utilize a range of antibiotic management strategies.

## 1. Introduction

The discovery of antibiotics is considered to be one of the most important of the twentieth century [1]. It revolutionized the way infections are treated [2] and, subsequent to the introduction of penicillin, a constant increase in antibiotic administration, among both medical and dental professionals [2], has been registered. This has led to the excessive and inappropriate use of antibiotics, which has unfortunately contributed to an increase in bacterial resistance to antimicrobials [3].

Antimicrobial resistance (AMR) is a global health problem that increases antibiotic treatment failure and, consequently, mortality and healthcare costs [4]. This phenomenon, secondary to mutational or genetic modifications of the various bacterial strains subjected to selective pressure, has led to a further increase in antibiotic prescription and use [5]. An alarming increase in antibiotic administration has been recorded globally as well as across Europe, resulting from excessive prescription rates not complying with antibiotic prescribing guidelines [6,7]. Between 30% and 66.5% of antibiotics prescriptions in medical settings are estimated to be unnecessary, and antibiotics are considered to be among the drugs most commonly prescribed in both dental outpatients and hospital inpatients [8,9]. Moreover, antibiotic overuse may also be ascribable to patients’ poor adherence to prescribed treatments, leading, in turn, to further antibiotic use. As a counterpart, antibiotic misuse may be secondary to patients’ self-administrations and inappropriate drugs prescriptions among clinicians [4]. Accordingly, antibiotic prescription in the dental practice, which is intended for prophylactic use against infective endocarditis in subjects with high-risk cardiac conditions [10,11,12], and for therapeutic use to treat odontogenic and non-odontogenic infections [13,14,15], should follow an accurate diagnosis and provide the most appropriate antibiotic at the recommended dose [16].

However, systemically delivered antibiotics were also found to be frequent and excessive in a 2018 Italian survey [17]. Consistently, antibiotic prescription in dentistry has generally increased in recent years [18], and dentists are still considered to be among the leading prescribers of systemically administered antibiotics [18]. Considering that Italy reached the ninth-highest systemic antibiotic prescription rate in Europe in 2017 [19], this cross-sectional study primarily aims to investigate, through a web-based survey among Italian dentists, the current antibiotic prescribing habits for prophylactic purposes based on dental procedures, and for therapeutic use according to dental diagnoses, in relation to dentists’ working experience, usual setting, and main practice. Moreover, although following antibiotic prescription guidelines is highly recommended [19] to help counteract AMR in dentistry [16], up to 66% of antibiotics prescribed by dentists were found to be not clinically indicated [17]. While Italy has among the highest levels of antimicrobial resistance for several bacterial species [20], no data are available concerning antibiotic prescribing attitudes towards the AMR phenomenon in Italian dental settings. Therefore, this study secondarily aims to assess the sources and frequency of consultation of the relevant guidelines, awareness and knowledge about antimicrobial resistance, and related antibiotic prescribing behaviors among Italian dentists.

## 2. Materials and Methods

### 2.1. Study Design

The present cross-sectional study was conducted by sending an online survey questionnaire to Italian dentists from 1 September to 31December 2021. The survey was created using an online survey development cloud-based software product called SurveyMonkey^®^ (SVMK, San Mateo, CA, USA).

### 2.2. Study Participants

The participants were approached via Facebook, WhatsApp, and mailing lists, and the sample size was estimated from similar studies [8].

Participation was voluntary and did not involve compensation. No personal health information was collected, the personal information of participants was protected, and there were no inhumane questions or investigations. All participants gave authorization to use their data, which were collected anonymously.

### 2.3. Data Collection

The questionnaire, developed after a literature review by a group of dentists with different dental specialties, consisted of 23 multiple-choice questions and two open questions, which were sorted into three main sections, specifically investigating:

1. Participants’ demographic features (their age and gender) and dental practice characteristics, concerning their working experience (in years), dental setting (whether they worked in a private practice alone, a private practice with associate(s), or whether they were a public employee, or a private employee), and their main practice (of oral surgery, endodontics, orthodontics, pediatric dentistry, periodontology, prosthetics/implantology, and others);
2. The antibiotic prescription habits of Italian dentists, for prophylaxis (in the case of simple tooth extractions, surgical tooth extractions, dental implant placement, periodontal regenerative surgery, implantology, and bone regenerative surgery) and for therapy (in the case of pulpitis, dental abscess, periodontitis, and pericoronitis), as well as their prescription frequency and the most frequent cause of prescribing systemic antibiotics;
3. Dentists’ attitudes towards antibiotics and AMR awareness, asking how often Italian dentists consult the guidelines for prescribing antibiotics, what the antibiotic resistance phenomenon is, dentists’ awareness of antibiotic resistance, prescribing behavior regarding antibiotics and the development of antibiotic resistance, the main reason why antibiotics are prescribed without indication or wrongly, and dentists’ main source of reference to access information on the topic of antibiotic resistance and antibiotic therapy.

### 2.4. Statistical Analysis

Frequencies and percentages for categorical data were computed. A chi-square test was used to assess the association between the general frequency of antibiotic prescription and gender, years of experience, and main activity; between the frequency of antibiotic prescription in the treatment and prophylaxis of various dental conditions and years of experience; and between knowledge of the antibiotic resistance phenomenon and years of experience. The main outcome was considered to be the association between wrong antibiotic prescription (for pulpitis) and the experience of the respondents. The effect size w and posthoc power of the study was calculated using G*Power software (version 3.1.9.7; Heinrich-Heine-Universität Düsseldorf, Düsseldorf, Germany). A standard statistical software package (SPSS, version 27.0; SPSS IBM, Armonk, NY, USA) was used. The level of significance was set at *p* < 0.05.

## 3. Results

Out of the 655 dentists that participated in the study, 382 (234 M, 148 F) completed the questionnaire.

### 3.1. Participants’ Demographic Features and Dental Practice

Out of the 655 dentists that participated in the study, 382 (234 M, 148 F) completed the questionnaire, giving a response rate of 58.32%. Participants’ demographic features and dental practice characteristics, detailed in Table 1, indicated that the majority of participants were men (60.9%) between 31 and 40 years of age (160/382, 41.7%), working as private practitioners, alone or in an association (348/382, 91.1%).

### 3.2. Antibiotic Prescription Habits of Italian Dentists

The antibiotic prescription habits of Italian dentists, investigated in the second section of the web-based survey and shown in Table 2, revealed that the main reasons for antibiotic prescriptions were dental abscesses (39.6%, 152/382), tooth extractions (24.5%, 94/382), and pulpitis (14.1%, 54/382).

In particular, 136/382 dentists (35.6%) prescribed antibiotic therapy in the case of pulpitis. The majority of those who prescribed antibiotics for pulpitis treatment (114/136, 83.8%) were dentists with less than 20 years of experience (*p* = 0.007, effect size w = 0.32, power = 99%). Moreover, 66/382 dentists (17.3%) always prescribed antibiotics as prophylaxis in simple tooth extractions. Most dentists (266/382) prescribed antibiotics “daily” (16.7%) or “weekly” (52.6%). Among the dentists with more than 20 years of experience, 72/86 (83.7%) prescribed antibiotics most frequently (“daily” or “weekly”) (*p* = 0.007). The dentists who most frequently prescribed antibiotics were those who mainly practiced oral surgery (72/74, 97.3%) and prosthetics/implantology (36/42, 85.7%), while those who less frequently (“monthly”, “annually”, or “never”) prescribed antibiotics were orthodontists (68/92, 73.9%) (*p* < 0.001). Antibiotics were prescribed “monthly”, “annually”, or “never” by 64/148 (43.2%) of female dentists compared to 54/236 (22.9%) of male dentists (*p* < 0.001). Men (90/116, 77.6%) made up a significantly higher proportion (*p* < 0.001) of oral surgeons and prosthetists/implantologists compared to women (26/116, 22.4%).

The majority of dentists (326) prescribed macrolides in the case of an allergy to penicillin, although 18 (4.7%) participants did not know which type of antibiotic to prescribe in such a scenario (Table 3).

### 3.3. Dentists’ Attitudes towards Antibiotics and Antimicrobial Resistance Awareness

The final section of the questionnaire assessed dentists’ attitudes towards antibiotics and antimicrobial-resistance awareness, respectively (Table 4).

The majority of dentists (378/382, 98.9%) reported being aware of the antibiotic resistance phenomenon, but only 28 (7.4%) of them consulted the guidelines for prescribing antibiotics.

Less than half of the participants (164/382, 42.9%) believed that their prescribing behavior could influence the development of antibiotic resistance. Less experienced dentists were more aware that their behavior had worsened the phenomenon of antibiotic resistance (*p* = 0.002).

The three main reasons as to why antibiotics were prescribed without indication or with an incorrect indication were as follows: to avoid capable litigation (34.8%), in the proximity of the weekend or a holiday when it was difficult to predict the evolution of the pathology (23.5%), and with non-compliant patients (23%).

Regarding behaviors that could favor the development of antibiotic resistance, 164 participants (42.9%) reported consulting the updated clinical guidelines, 88 (23%) reported obtaining the information from scientific magazines and/or scientific journals, and 36 (9.4%) reported reading forums on the internet.

## 4. Discussion

This cross-sectional web-based survey study aimed to investigate the current antibiotic prescribing habits for prophylactic use based on dental procedures, and for therapeutic purposes according to dental diagnoses, in relation to dentists’ working experience, usual setting, and main practice. The secondary aim was to assess the sources and frequency of the consultation of the relevant guidelines, awareness and knowledge about antimicrobial resistance, and related antibiotic prescribing behaviors among Italian dentists.

### 4.1. Antibiotic Prescription Habits of Italian Dentists

Among the study participants, female dentists tended to prescribe antibiotics less frequently, prescribing antimicrobials “monthly”, “annually”, or “never” in 43.2% of cases, compared to male participants. Similarly, 73.9% of the orthodontists selected “monthly”, “annually”, or “never” with regard to antibiotic prescription in their ordinary practice. Conversely, those dentists mainly practicing prosthetics/implantology and oral surgery reported higher rates of antimicrobial administration, with 85.7% and 97.3% reporting “daily” or “weekly” prescriptions, respectively. Such findings may be explained by the fact that, among the respondents who were prosthetists/implantologists and oral surgeons, men made up a significantly higher proportion than women. In addition, the highest frequency of antibiotic prescription was recorded among dentists with >20 years of dental practice, excluding for prophylactic purposes in simple tooth extractions and for therapeutic use in the case of pulpitis, in which less experienced dentists prescribed antibiotics at a higher frequency. This was probably because they are less confident in their ability to deal with possible complications.

The presented results show that many dentists prescribed antibiotics for both prophylactic purposes and therapeutic use in several clinical situations, even if not indicated in the guidelines. However, a general overuse of antibiotics, both for prophylactic and therapeutic purposes, was noted in the present study. This is in line with findings from other countries that also attest to an increase in dental antibiotic use, such as France [21], the Czech Republic, which recorded a 60% increase from 2006 to 2012 [22], and Canada, which recorded a 62% increase from 1993 to 2013 [23]. Contrarily, a reduction in overall antimicrobial administration was recorded in Australia [23] and in the UK, the latter seeing a 26.8% reduction from 2014 to 2018 [24].

Prophylactic antibiotic administration to healthy patients was reported by approximately half of the participants, in cases of dental implant placement (57.1%), regenerative surgery (52.4%), and surgical tooth extractions (47.6%). It is worth noting that 17.3% of dentists in the present survey reported administering antimicrobials to healthy patients in cases of simple tooth extractions. Similar findings were described in several other studies, indicating that dentists often overprescribe antibiotics for prophylaxis, even in healthy patients undergoing invasive dental procedures, such as surgical extractions, dental implant positioning, and endodontic procedures, without observing the relevant guidelines [14,25]. It should be noted that guidelines and recommendations are still lacking for immunocompromised subjects, such as those suffering from type 1 diabetes, HIV infection or AIDS, although increasingly required; thus, antibiotic prescription for prophylactic purposes is justified according to the American Dental Association [26].

Antibiotic administration for therapeutic use in healthy patients was reported by 84.3% of dentists, specifically in cases of dental abscesses, followed by pericoronitis (58.6%), periodontitis (31.9%), and pulpitis (19.4%). In particular, 35.6% of the participants prescribed antibiotics for therapeutic use in cases of pulpitis, despite the evidence being considered insufficient to support the use of antibiotics for the treatment of irreversible pulpitis and periapical abscesses [9,27]. Analogous results were obtained from a study conducted in Spain in which antibiotics were prescribed in cases of irreversible pulpitis by 40% of endodontists [28]. Contrarily, in Belgium, antibiotic administration was reported in only 4.4% of cases with pulpitis [29]. Indeed, the use of systemic antibiotics has been shown to be unnecessary in irreversible pulpitis, necrotic pulps, and acute apical abscesses, unless a rapid spread of the odontogenic infection with systemic involvement becomes evident. The treatment of choice for pulpitis is pulpectomy, and antimicrobial administration should only be utilized in cases of systemic complications, as described in the guidelines [1,9,14].

### 4.2. Dentists’ Attitudes towards Antibiotics and Antimicrobial Resistance Awareness

Italian dentists often reported ignoring the guidelines, which is one of the main reasons underlying inappropriate antibiotic prescription. In this study, only 7.4% of dentists reported always consulting the current antimicrobial prescription guidelines, while the majority consulted the guidelines “seldomly” (48%) or “never” (5.3%). From this perspective, it is crucial to raise awareness of AMR among Italian dentists, as it is a serious global public health problem and is directly related to increased healthcare costs and poorer clinical outcomes. Awareness-raising programs should focus on understanding how important it is to consult the updated guidelines in order to combat this phenomenon [23]. The main sources consulted to obtain information on the AMR phenomenon and antibiotics administration recommendations were the relevant guidelines (42.9%), followed by the Internet (9.4%), dedicated courses (8.9%), and colleagues (6.3%). Given the heterogeneity of the reported sources, improving dentists’ access to all of them and enhancing inter-professional collaboration may be beneficial. Three hundred seventy-eight (98.9%) dentists declared being aware of the antimicrobial resistance, mainly characterizing it as a growing phenomenon (91.1%). However, only 42.9% of participants declared considering incorrect prescribing behavior with regard to AMR development, clearly highlighting the need for a wider diffusion of such information.

The participants in the present study reported prescribing antibiotics to avoid capable litigation (34.8%) or in cases of patients with poor compliance to dental treatments (23%). In this respect, the COVID-19 pandemic probably increased antibiotic prescriptions due to the impossibility of making an accurate diagnosis during the lockdown and patients’ subsequent fear of returning to a dental practice [30].

Similar to our results, overuse and misuse of antibiotics in dentistry was registered in France [21], the USA [25], and the UK, where 37.6% of adults and the 39.0% of pediatric patients attending dental clinics for emergencies were administered antimicrobials and, in 57.4% of cases, antibiotics were prescribed even if no diagnosis was documented [31,32,33,34]. This is also consistent with findings from a cross-sectional study conducted in India, which revealed that 35% of dentists prescribed antibiotics prior to or instead of starting local treatment [31,32].

To avoid the overuse and misuse of antibiotics, several factors should be considered before commencing treatment. These include clinical indications for antibiotic administration, the patient’s oral health status, comorbidities, current medication status, and, if possible, a microbiological test to correctly determine the risk/benefit ratio related to systemically delivered antibiotic administration [29]. Moreover, the shortest effective course of a narrow-spectrum antibiotic should be preferred, and the patient should be monitored for the entire course of therapy; hence, amoxicillin and phenoxymethylpenicillin are recommended as the first-choice therapeutic antibiotics in patients without a penicillin allergy [35,36,37]. In patients with a penicillin allergy, macrolides were the most frequently prescribed antibiotics (83.4%), followed by lincosamides(4.2%), as shown in Table 3. Macrolides were also preferred by 52.9% of Italian oral implant surgeons [25], as revealed by another recent cross-sectional survey. Conversely, Baudet et al. [21] found that, among French dentists, clindamycin was the most frequently prescribed antibiotic (46.7%) in patients allergic to penicillin, followed by spiramycin + metronidazole (32.2%), even though both administrations (clindamycin and a spiramycin + metronidazole fixed-dose combination) may not be appropriate for patients who are allergic to penicillin [26]. Clindamycin was similarly shown to be the most prescribed antibiotic in penicillin-allergic patients in Spain (63.2%) [27,28] and the USA (95.4%) [35,36], where dentists are among the leading prescribers of antibiotics and often use broad-spectrum antibiotics for longer than necessary, even in cases in which it is not necessary or compliant with the guidelines [25].

Along with appropriate short-term antibiotics prescriptions in dentistry, timely management of oral, dental, and periodontal dysbioses is strongly advocated, with adequate dental procedures and antiseptic use which may enhance the control of infections [11,14]. Indeed, a body of evidence supports the inter-connections between the oral, dental, and periodontal microbiome, reaching distant organs through the systemic bloodstream and by microaspiration, potentially causing infections [14,38,39,40,41,42]. These may be especially relevant for healthcare-associated infections, mainly caused by multidrug-resistant bacteria, generally associated with an increased length of hospitalization, morbidity, mortality, and healthcare costs [38,39,40,41,42,43].

Therefore, multiple combined strategies are needed to counteract antimicrobial resistance in dental settings, improving awareness and knowledge of AMR, optimizing antibiotics use, and strengthening surveillance [17,44].

The present study has various limitations, for example, the questionnaire was self-administered, and so specific questions may have been improperly interpreted, although it may be conceivable that self-administration permits more authentic answers that are truly representative of the respondent’s beliefs. In addition, an exact response rate could not be computed. However, this study assessed antibiotic administration among Italian dentists not only for prophylactic purposes [8], but also therapeutic use, evaluating it in relation to dentists’ working experience, usual setting, and main practice. Moreover, different from other studies, the present survey specifically focused on dentists who voluntarily participated [45]. Furthermore, Italian dentists’ awareness of antimicrobial resistance was investigated along with their consultation of the relevant guidelines, highlighting the need to spread relevant information to dental healthcare workers and patients widely. This finding might be especially relevant considering that dental settings are generally private in Italy, dentists usually work alone, and subjects receiving dental care are most frequently outpatients. Coherently, further studies should be performed to investigate patient behaviors that may negatively affect the AMR phenomenon, such as non-adherence to treatment and non-medical prescriptions.

## 5. Conclusions

This study, conducted among Italian dentists, showed a similar trend to that seen in other countries that have a high prevalence of overuse and misuse of systemic antibiotics in dental practice. In addition, it highlights the need to enhance antimicrobial-resistance awareness among Italian dentists.

Antimicrobial stewardship should be deeply integrated in dental practice and oral health care. Moreover, through education and training, practitioners’ antibiotic administration should be improved in order to reduce the number of unnecessary prescriptions, and patients’ adherence to antimicrobial treatment should be enhanced in order to limit non-medical prescriptions.

## Figures and Tables

**Table 1:** Personal characteristics of dentists and their work habits.

		Frequency	Percentage
Gender	M	234	60.9%
	F	146	38%
	Other	2	1%
Age (years old)	<30	80	20.8%
	31–40	160	41.7%
	41–50	62	16.1%
	51–60	56	14.6%
	>61	24	6.8%
Working Experience (years)	<2	54	14.1%
	2–10	136	35.4%
	11–20	106	27.6%
	21–30	44	11.5%
	>30	42	11.5%
Dental setting	Private practice alone	192	50.5%
	Private practice with associate(s)	156	40.6%
	Public employee	14	3.6%
	Private employee	20	5.2%
Main practice	Oral surgery	74	19.3%
	Endodontics	126	33.3%
	Orthodontics	92	24%
	Pediatric dentistry	6	1.6%
	Periodontology	18	4.7%
	Prosthetics/Implantology	42	10.9%
	Others	24	6.2%

**Table 2:** Prescription habits of Italian dentists.

		Frequency	Percentage
Antibiotic prophylaxis in simple tooth extractions	Always	66	17.3%
	Only in unhealthy patients	172	45%
	Never	128	33.5%
	I don’t do it	16	4.2%
Antibiotic prophylaxis in surgical tooth extractions	Always	182	47.6%
	Only in unhealthy patients	152	39.8%
	Never	28	7.3%
	I don’t do it	20	5.2%
Antibiotic prophylaxis for dental implant placement	Always	218	57.1%
	Only in unhealthy patients	52	13.6%
	Never	24	6.3%
	I don’t do it	88	23%
Antibiotic prophylaxis in regenerative surgery	Always	200	52.4%
	Only in unhealthy patients	44	11.5%
	Never	22	5.8%
	I don’t do it	116	30.4%
Antibiotic therapy in implantology and regenerative surgery	Always	218	57.1%
	Only in unhealthy patients	50	13.1%
	Never	8	2.1%
	I don’t do it	106	27.7%
Antibiotic therapy in pulpitis	Always	74	19.4%
	Only in unhealthy patients	62	16.2%
	Never	214	56%
	I don’t do it	32	8.4%
Antibiotic therapy in dental abscess	Always	322	84.3%
	Only in unhealthy patients	30	7.8%
	Never	16	4.2%
	I don’t do it	14	3.7%
Antibiotic therapy in periodontitis	Always	122	31.9%
	Only in unhealthy patients	98	25.6%
	Never	118	30.9%
	I don’t do it	44	11.5%
Antibiotic therapy in pericoronitis	Always	224	58.6%
	Only in unhealthy patients	72	18.8%
	Never	52	13.6%
	I don’t do it	34	8.9%
How often do you prescribe systemic antibiotics?	Daily	64	16.7%
	Weekly	202	52.6%
	Monthly	82	21.3%
	Annually	20	5.2%
	Never	16	4.2%
The most frequent cause of prescribing systemic antibiotics	Pulpitis	54	14.1%
	Periodontal abscess	10	2.6%
	Implantology	30	7.8%
	Dental abscess	152	39.6%
	Peri-implantitis	6	1.6%
	Extraction	94	24.5%
	Oral surgery	22	5.7%
	Regenerative surgery	10	2.6%
	Other	6	1.6%

**Table 3:** Type of antibiotic prescribed in case of penicillin allergy.

Antibiotics	
Macrolides	326
Lincosamides	16
Fluoroquinolones	8
Cephalosporin	8
Metronidazole	2
Vancomycin	2
Tetracycline	2
Does not know	18

**Table 4:** Knowledge of the phenomenon of antibiotic resistance.

		Frequency	Percentage
How often Italian dentists consult guidelines for prescribing antibiotics?	Always	28	7.4%
	Often	148	39.1%
	Occasionally	192	48.1%
	Never	20	5.3%
Dentists know what the antibiotic resistance phenomenon is?	Yes	378	98.9%
	No	4	1.1%
Antibiotic resistance is…	A growing phenomenon	348	91.1%
	An upcoming phenomenon	18	4.7%
	A phenomenon eradicated	4	1.1%
	A phenomenon described in the scientific literature	12	3.1%
Prescribing behavior regarding antibiotics and development of antibiotic-resistances	Yes	164	42.9%
	No	140	36.7%
	Don’t know	78	20.4%
The main reason why antibiotics are prescribed without indication or wrongly	Non-compliant patients	86	23%
	Patient who is unknown	36	9.6%
	To avoid capable litigation	130	34.8%
	In the proximity of the weekend/festivity ifit is difficult to predict the evolution of the pathology	88	23.5%
	The patient asks to return to work quickly	10	2.7%
	For pathologies of a viral or fungal nature	24	6.4%
The main source consulted to obtain information on antimicrobial resistance and antibiotics administration	Forum on the internet	36	9.4%
	Textbooks	20	5.2%
	Scientific magazines	88	23%
	Consultation of the guidelines	164	42.9%
	Direct communication with referring colleagues	24	6.3%
	FAD courses	34	8.9%
	Scientific information from the pharmaceutical industry	16	4.2%

## Data Availability

Data are available upon request to the contact author.
